# Optogenetic interrogation reveals separable G-protein-dependent and -independent signalling linking G-protein-coupled receptors to the circadian oscillator

**DOI:** 10.1186/s12915-017-0380-8

**Published:** 2017-05-15

**Authors:** Helena J. Bailes, Nina Milosavljevic, Ling-Yu Zhuang, Elliot J. Gerrard, Tomoki Nishiguchi, Takeaki Ozawa, Robert J. Lucas

**Affiliations:** 1 Faculty of Biology, Medicine and Health, The University of Manchester, Manchester, UK; 2 Department of Chemistry, The University of Tokyo, Tokyo, Japan

## Abstract

**Background:**

Endogenous circadian oscillators distributed across the mammalian body are synchronised among themselves and with external time via a variety of signalling molecules, some of which interact with G-protein-coupled receptors (GPCRs). GPCRs can regulate cell physiology via pathways originating with heterotrimeric G-proteins or β-arrestins. We applied an optogenetic approach to determine the contribution of these two signalling modes on circadian phase.

**Results:**

We employed a photopigment (JellyOp) that activates Gαs signalling with better selectivity and higher sensitivity than available alternatives, and a point mutant of this pigment (F112A) biased towards β-arrestin signalling. When expressed in fibroblasts, both native JellyOp and the F112A arrestin-biased mutant drove light-dependent phase resetting in the circadian clock. Shifts induced by the two opsins differed in their circadian phase dependence and the degree to which they were associated with clock gene induction.

**Conclusions:**

Our data imply separable G-protein and arrestin inputs to the mammalian circadian clock and establish a pair of optogenetic tools suitable for manipulating Gαs- and β-arrestin-biased signalling in live cells.

**Electronic supplementary material:**

The online version of this article (doi:10.1186/s12915-017-0380-8) contains supplementary material, which is available to authorized users.

## Background

Circadian rhythms are generated by cell-autonomous molecular oscillators widely distributed across the mammalian body. In order to perform their function of providing temporal order to physiological processes, these independent oscillators need to be synchronised among themselves and with diurnal rhythms in the environment associated with the earth’s axial rotation. Among the signalling molecules known to influence the clock are several that engage G-protein-coupled receptors (GPCRs). Accordingly, GPCRs and their downstream signalling cascades are known to regulate the expression of elements of the molecular oscillator and the phase and/or period of the clock [1–7].

GPCRs engage numerous cellular signalling pathways and second messenger systems. The best characterised of these are those downstream of the heterotrimeric G-proteins that are activated by GPCRs and in turn alter the activity of numerous effector enzymes. More recently, however, a quite separate signalling activity involving β-arrestins has been described. β-arrestins bind phosphorylated residues in the C-terminal intracellular tail of activated GPCRs. Their long established role is to terminate G-protein binding and thus quench signalling. Binding of arrestin also facilitates internalisation and recycling of activated receptors. The signalling activity of β-arrestins is related to their ability to act as scaffolds, facilitating the phosphorylation of numerous proteins and changing the activity of intracellular kinase cascades (ERK, AKT, PI3, p38, RhoA [8, 9]). In this way, they are able to influence varied aspects of cell physiology, ranging from modifications of the cytoskeleton, to changes in gene expression at the level of both transcription and translation [10].

Here, we set out to address the question of the extent to which GPCR inputs to the circadian clockwork can employ both G-protein-dependent and -independent signalling pathways. Involvement of the former is implied by the observation that pharmacological manipulation of second messenger systems downstream of G-protein activation successfully changes clock gene expression and shifts the clock. To date, there is no evidence that arrestin pathways are also employed.

A challenge in studying influences on the circadian clock is that the clock’s response to incoming signals typically depends upon the clock phase at which they arrive. Thus, the same signal can either have no impact or can delay or advance clock phase depending upon when it appears. This phenomenon is described by a ‘phase response curve’ and is necessary if the clock is to be synchronised to periodic inputs. In the laboratory, this means that experimental manipulations probing mechanisms of entrainment should be carefully timed with respect to clock phase. Optogenetics represents an attractive method of achieving this. The mammalian clock (at least outside of the retina) is not directly photosensitive, allowing light to be used to control optogenetic actuators with high temporal fidelity. Accordingly, photopigments driving light dependent changes in membrane potential and intracellular calcium have been used to explore the ability of time-delimited manipulations in these aspects of cell physiology to shift the clock [11, 12]. Here, we adopted this approach by employing an opsin photopigment (JellyOp) that is naturally coupled to a Gαs signalling cascade driving increases in cAMP [13, 14]. Gαs-coupled GPCRs are targets for signalling molecules known to shift the clock [2, 6], and pharmacological increases in cAMP represent a strong phase resetting stimulus [15, 16]. It is less clear whether any arrestin-dependent signalling of JellyOp could influence the clock. To address this possibility, we introduce a point mutation (F112A) that inhibits JellyOp’s ability to activate G-protein but retains its light-dependent arrestin interaction. We show that both native JellyOp and JellyOp F112A support light-dependent shifts in the fibroblast circadian clock, but do so at different circadian phases and via different molecular mechanisms. These experiments thus reveal separable G-protein-dependent and -independent pathways through which GPCRs can entrain circadian clocks.

## Results

### Optogenetic activation of Gαs signalling

We set out initially to determine an optimal optogenetic approach to achieve time-delimited activation of Gαs signalling. Two photopigments have been applied to Gαs signaling in the literature, both based upon metazoan opsins that are naturally light activated GPCRs. The first is a chimeric receptor termed Opto-β2AR, in which the photosensitive core of bovine rod opsin was fused with intracellular domains from the hamster β_2_ adrenergic receptor [17, 18]. Rod opsin is naturally Gαt coupled, but Opto-β2AR attains the Gαs signalling ability of the β_2_ adrenergic receptor. The other approach is to employ JellyOp, a photopigment from the box jellyfish *Carybdea rastonii* that is naturally Gαs coupled [13, 14]. We have previously reported that the JellyOp pigment better supports responses to repeated light exposure in cell culture and especially in the absence of cis isoforms of retinaldehyde [13, 14]. To further determine which of these provides the highest level of control we undertook side-by-side comparison of other features of the optogenetic response (baseline activity, photosensitivity and G-protein selectivity) provided by a humanised version of the Opto-β2AR (hereafter referred to as Opto-hβ_2_AR, in which bovine and hamster sequences were replaced with equivalent residues from human genes) and JellyOp in HEK293 cells expressing a luminescent reporter for intracellular cAMP (GloSensor 22 F; Promega Corp).

**Fig. 1:**
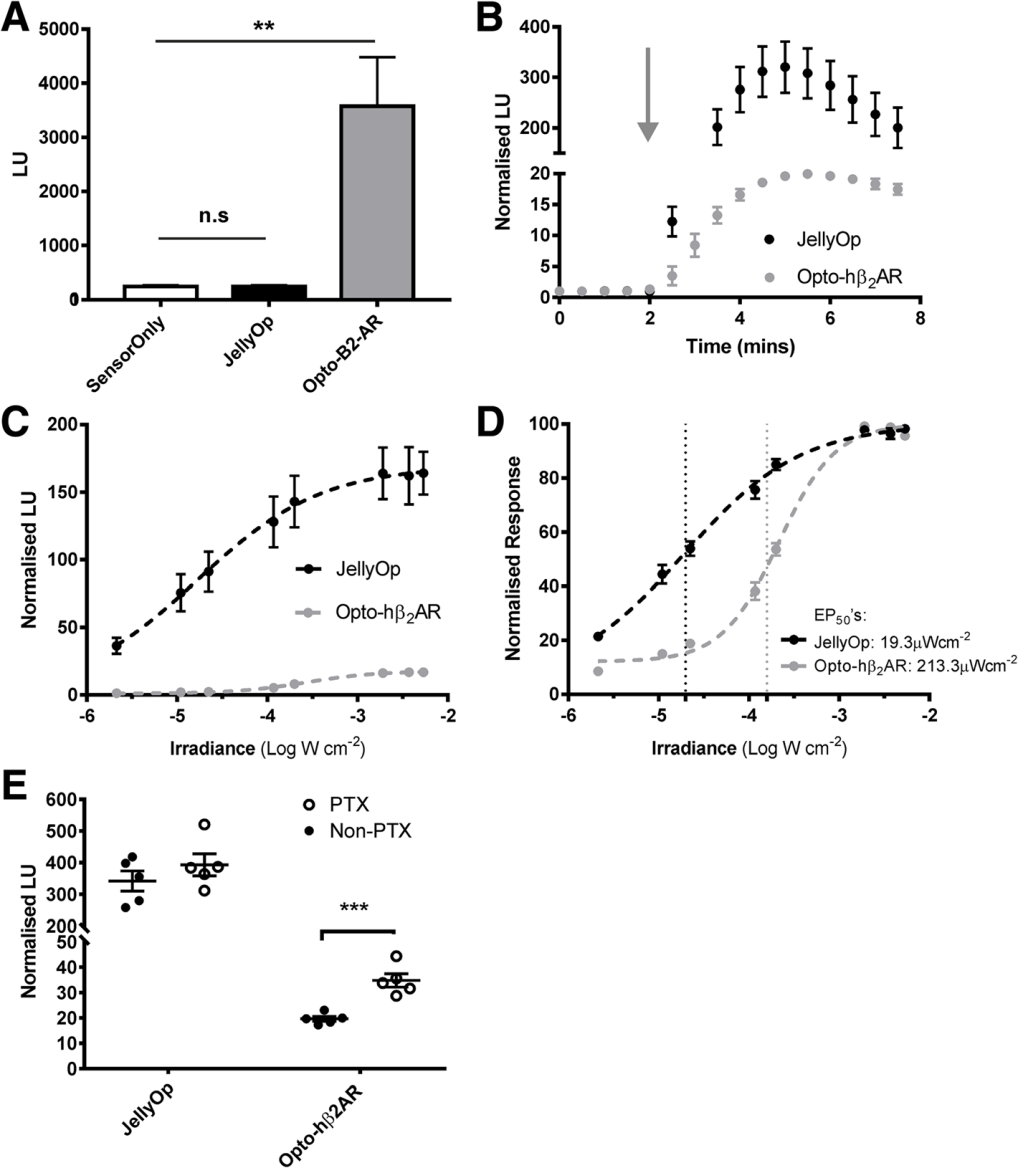
Comparison of JellyOp and Opto-hβ_2_AR for optogenetic control over Gαs signalling. **a** Baseline (dark) luminescence of cells expressing JellyOp, Opto-hβ_2_AR or the GloSensor reporter alone. Opto-hβ_2_AR causes a significant increase in baseline luminescence compared to the GloSensor alone condition (**P < 0.01, one-way ANOVA with Dunnett’s multiple comparison, mean ± SEM, n = 3; values are reported in Additional file 1). **b** Time course showing changes in cAMP reporter luminescence in cells expressing either JellyOp or Opto-hβ2AR held in the dark and given a 5 second light flash (λ_max_ = 470 nm, 3 mW cm^–2^) at 2 min, as indicated by a grey arrow (data expressed as raw luminescence normalised to mean luminescence over baseline recording, mean ± SEM, n = 5; values in Additional file 1). **c** Irradiance response curves for the peak increase in cAMP reporter luminescence (normalised to baseline) produced by the two photopigments (light: 2 s, λ_max_ = 470 nm). Data fitted with standard sigmoidal dose response curves; mean ± SEM, n = 3. **d** Irradiance response profiles from (**c**) expressed as a percentage of maximum luminescence response for each photopigment, fitted with sigmoidal dose response curves (dotted lines mark irradiance at 50% max response for JellyOp and Opto-hβ2AR, mean ± SEM, n = 3; values in Additional file 1). **e** Comparison of peak increase in cAMP reporter luminescence (normalised to baseline) following a saturating light pulse (as in (**b**)) with and without pertussis toxin (10 μM PTX). The increase in response amplitude in Opto-hβ2AR expressing cells following PTX administration implies that significant Gαi/o activation by this receptor had suppressed the response in the absence of PTX (*P* < 0.001, max response amplitude 19.65 ± 0.97 vs. 34.8 ± 2.64, without/with PTX; Student’s *t* test, n = 5). This effect is absent in JellyOp expressing cells (*P* = 0.31, 341.7 ± 31.6 vs. 392.96 ± 34.5 without/with PTX, max response amplitude, Student’s *t* test n = 5; values in Additional file 1). All data mean ± SEM unless stated

Live cell recording revealed that transient expression of Opto-hβ_2_AR, but not JellyOp, significantly elevated baseline cAMP luminescence in the dark (*P* < 0.01 vs. cells expressing GloSensor but not photopigment, one-way ANOVA with Dunnett’s multiple comparisons, mean of 10 cycles in the dark, n = 5; Fig. 1a; values in Additional file 1). A 5 second pulse of blue light (λ_max_ 470 nm, 3 mW cm^–2^s^–1^) induced an increase in luminescence in cells expressing either pigment, but response amplitude was an order of magnitude greater in cells expressing JellyOp than Opto-hβ_2_AR (*P* < 0.01, max response amplitude, unpaired Student’s *t* test, n = 5; Fig. 1b; values in Additional file 1). This difference in amplitude was retained across a range of light pulse intensities (Fig. 1c; values in Additional file 1). Responses were next normalised to the maximum for that pigment in each run of the assay, to account for the difference in absolute response amplitude between the two pigments and for inter-assay variability. Data processed in this way revealed a significant reduction in photosensitivity between the two pigments (*P* < 0.001, EP_50_ = 19.27 ± 1.34 and 213.3 ± 12.28 mW cm^–2^ for JellyOp and Opto-hβ_2_AR, respectively, mean ± SEM, unpaired Student’s *t* test, n = 3; Fig. 1d; values in Additional file 1). One potential explanation for the reduced cAMP response of the Opto-hβ2AR is that it retains significant Gαi/o activity, as is the case for the β2 adrenergic receptor itself [19]. In HEK293 cells, Gαs/Gαi/o pathways have antagonistic effects on cAMP [13]. In accordance with this hypothesis, application of the Gαi inhibitor pertussis toxin (PTX) led to a significant increase in the amplitude of the Opto-hβ2AR light response (*P* < 0.001, Max response amplitude: 19.65 ± 0.97 vs. 34.8 ± 2.64 for non-PTX- vs. PTX-treated cells, mean ± SEM, Student’s *t* test, n = 5; Fig. 1e; values in Additional file 1). This indicates that the Opto-hβ2AR has significant Gαi/o signaling activity. There was no effect of PTX on the light response of JellyOp expressing cells (*P* = 0.31, Student’s *t* test, n = 5, Fig. 1e; values in Additional file 1) indicating that this pigment has good selectivity for the Gαs over Gαi/o pathway. Even after PTX treatment, the Opto-hβ2AR light response was qualitatively deficient compared to that produced by JellyOp (Fig. 1d), revealing that, while part of JellyOp’s improved performance in this assay can be attributable to higher selectivity for Gαs it must also have other, more generic, advantages.

### JellyOp causes light-dependent cAMP induction and circadian phase shifts in fibroblasts

**Fig. 2:**
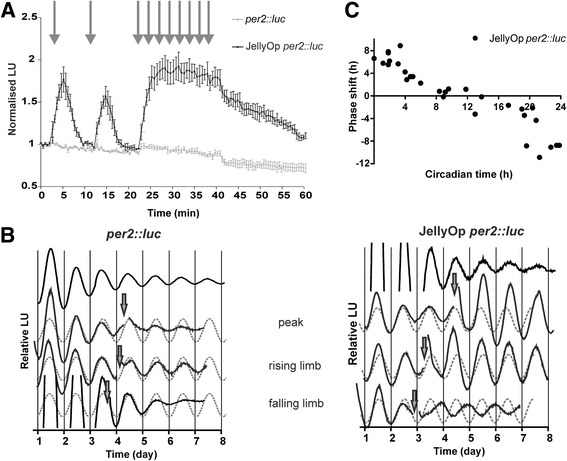
JellyOp allows time-dependent manipulation of the circadian clock. **a** JellyOp-expressing *per2::luc* rat1 fibroblasts (black squares and line) show a robust increase in luminescent cAMP reporter activity following exposure to light, which is absent in cells expressing only the GloSensor reporter (grey squares and line). The data present representative traces of baseline normalised luminescence units (mean ± SEM) from triplicate samples in a single assay. Cells were pulsed with single light flashes at 2 and 12 min, followed by 30 light flashes (at 30 second intervals) starting at 22 min, indicated by grey arrows. **b** The circadian *luciferase* rhythm of *per2::luc* (on the left) and JellyOp-expressing *per2::luc* (on the right) rat1 fibroblasts exposed to 4 h of intermittent white light (28.40 mW cm^–2^) at various phases of the *per2* rhythm (indicated by arrows: at CT11.2, CT2.1, CT17.3 for *per2::luc* and at CT11.8, CT3.1, CT19.6 for JellyOp *per2::luc*). For phase analysis, baseline corrected bioluminescence rhythms prior to light treatment were modelled by a sine wave (grey dotted line). Time of first trough in *per2::luc* rhythm aligned for all traces to facilitate comparisons. **c** Phase response profile for JellyOp-expressing *per2::luc* rat1 fibroblasts illustrates the relationship between the timing of light onset and magnitude of phase shifts in *per2* rhythm. Data represent the extent of phase shifts captured from individual light pulsed cultures and plotted on a y-axis where positive and negative values denote phase advances and delays, respectively (values are reported in Additional file 1)

**Fig. 3:**
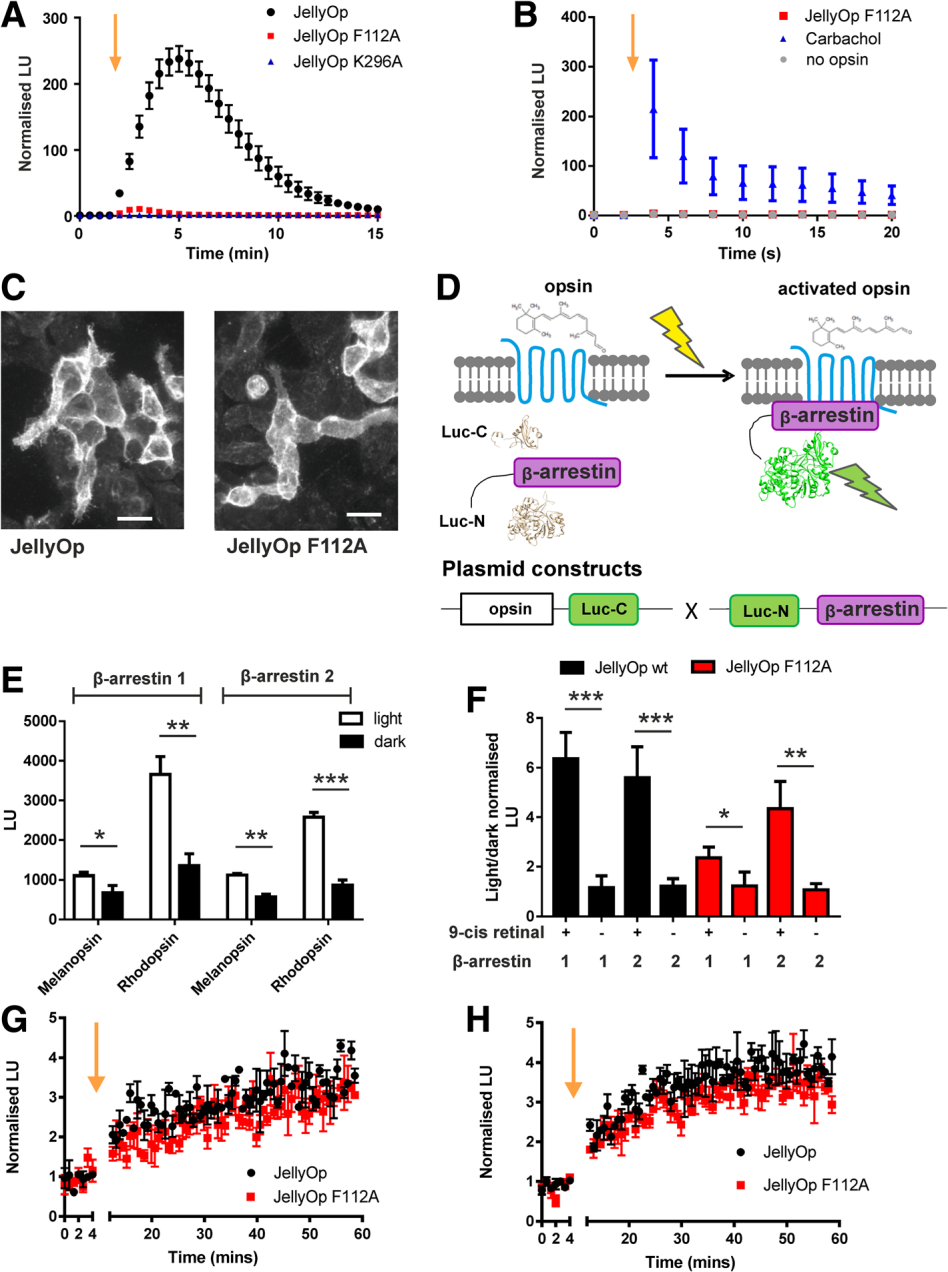
Characterisation of signalling properties of Gαs-coupled JellyOp and its structural arrestin-biased mutant JellyOp F112A. **a** HEK293 cells expressing JellyOp F112A mutant (red squares) show a much reduced increase in luminescent cAMP reporter activity following exposure to a flash of light at 2 min (yellow arrow) compared to cells expressing JellyOp (black circles, n = 6). The response of cells expressing an inactivation mutant (K296A bovine numbering; blue triangles) are shown for comparison (n = 3; values in Additional file 1). **b** A significant increase in aequorin calcium reporter luminescence is seen in response to carbachol (100 μM) treatment of HEK293 cells (blue triangles, n = 5). No calcium reporter increase above background is seen in cells expressing JellyOp F112A (red squares) and exposed to light (*P* > 0.05, *t* test, n = 4; values in Additional file 1). **c** Fluorescence immunohistochemical labelling of the 1D4 epitope in HEK293 cells transiently expressing JellyOp (panel on the left) and F112A mutant (panel on the right). Scale bar: 20 μm. **d** A schematic presentation of an arrestin-based luminescent probe. Two fragments of a luciferase were fused to an opsin (Luc-C) and β-arrestin (Luc-N), respectively. When opsins are activated, β-arrestin is recruited to the receptors and the fragments recover their luminescence property. **e** β-arrestin luminescent assay reveals that light stimulation significantly enhances luminescence for β-arrestin 1 and 2 in HEK293 cells expressing positive control opsins known to have light-induced arrestin interaction (melanopsin and rhodopsin; n = 4 biological replicates; values are reported in Additional file 1). **f** β-arrestin luminescent assay reveals light triggered luminescence induction in JellyOp and F112A mutant only in the presence of 9-cis retinal. (*P < 0.5, **P < 0.1, ***P < 0.01, Student’s *t* test, n = 4; values in Additional file 1). **g**–**h** Kinetics of JellyOp (black circles and line) and F112A mutant (red square and line) interaction with β-arrestin 1 (**g**) and β-arrestin 2 (**h**) after a light flash (3 mW cm^–2^) indicated by a yellow arrow (n = 3; values in Additional file 1)

**Fig. 4:**
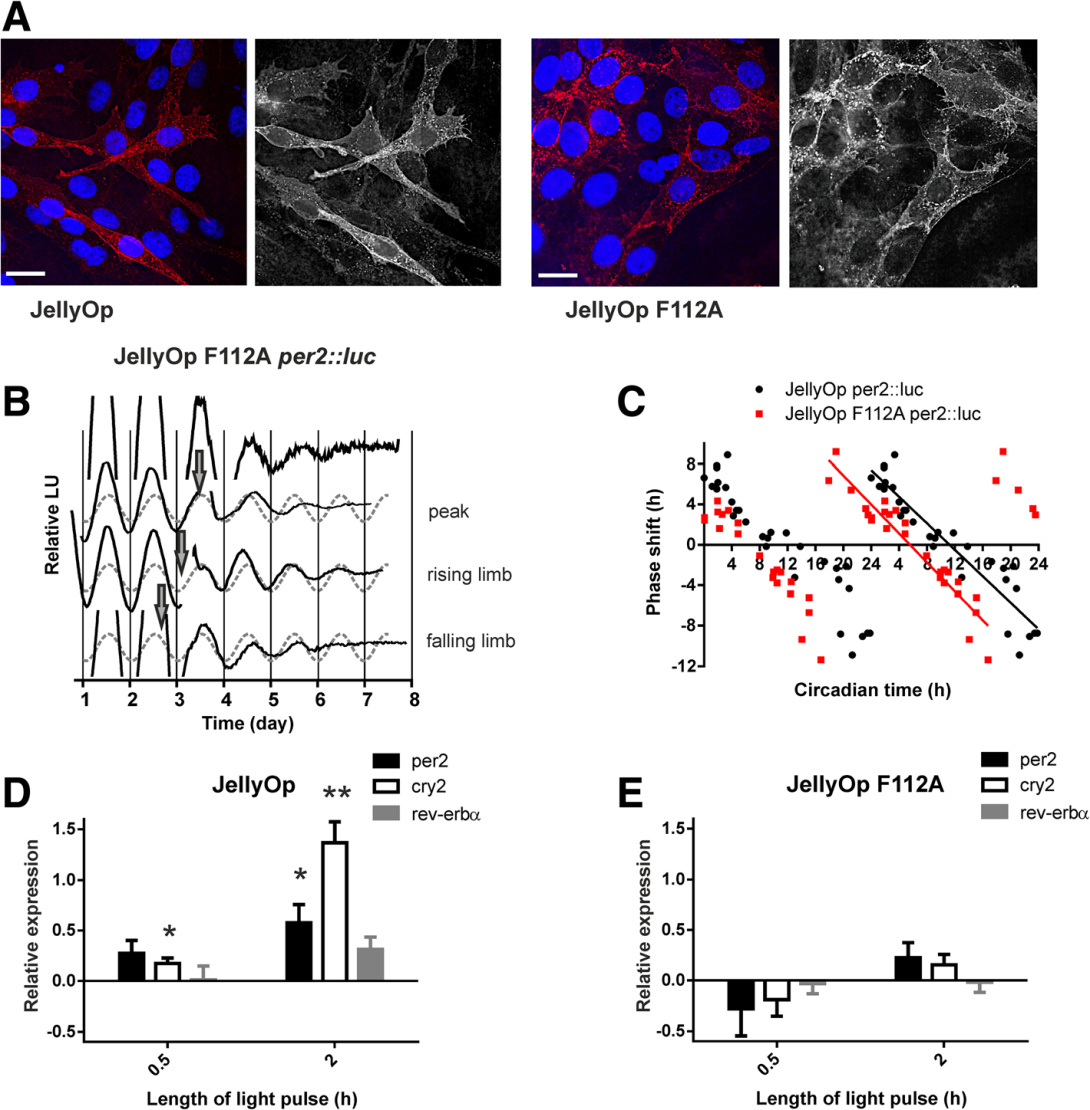
Phase shifts of the fibroblast clock by the arrestin-biased JellyOp F112A mutant. **a** Immunocytochemistry photomicrographs showing detection of JellyOp variants stably expressed in rat1 fibroblasts and labelled with a 1D4 antibody (pseudo coloured red in the merged image on the left; monochromatic image on the right). Cells are also stained with DAPI (blue). Scale bar = 20 μm. **b** The circadian *per2* rhythm of JellyOp F112A-expressing *per2::luc* rat1 fibroblasts exposed to 4 h intermittent white light (28.40 mW cm^–2^) at various phases of the *per2* rhythm (indicated by arrows at CT12.5, CT2.6, CT17.9). For phase analysis, baseline corrected bioluminescence rhythms prior to light treatment were modelled by a sine wave (grey dotted line). Time of first trough in per2:luc rhythm aligned for all traces to facilitate comparisons. **c** Double-plotted phase response profile for JellyOp (black circles, replotted from Fig. 2c) and JellyOp F112A (red squares) expressing *per2::luc* rat1 fibroblasts, illustrates the relationship between the timing of light onset and magnitude of phase shifts in *per2* rhythm. Data represent extent of phase shifts captured from individual light pulsed cultures and plotted on a y-axis where positive and negative values denote phase advances and delays, respectively. Linear regression fits with R^2^ of 0.88 and 0.91 for JellyOp and JellyOp F112A, respectively (values in Additional file 1). **d** Acute light-dependent changes in transcription of core clock genes of JellyOp (**d**) and JellyOp F112A (**e**). mRNA was extracted from fibroblasts exposed to 30 min or 2 h intermittent white light (28.40 mW cm^–2^), at the circadian time when the biggest phase shift was produced for each optogenetic tool, namely at CT2 and CT20 for JellyOp (**d**) and JellyOp F112A (**e**), respectively. *per2* and *cry2* are upregulated in JellyOp wt and both have cAMP responsive element (*CRE*) in their promoters that regulates their expression. All results were normalised to actin expression. All data are presented as the relative gene expression mean ± SEM (*P < 0.05, **P < 0.01, One-sample *t* test, n = 4; values in Additional file 1)

Based upon the results of the side-by-side comparison of JellyOp and Opto-hβ_2_AR, we chose the former for the remaining experiments in this study. As HEK293 cells do not exhibit circadian oscillations, we switched to a fibroblast cell line (rat1) that does. We employed a version of this line that carries a luciferase coding sequence under control of a minimal Period2 (*Per2*) promoter (*per2::luc* [20]). *Per2* is a component of the molecular circadian clock whose expression shows strong circadian regulation. As a result, the phase of circadian clocks in the rat1 fibroblast reporter line can be tracked in real time by measuring luminescence. We first confirmed that JellyOp can induce cAMP in the rat1 fibroblasts by creating a cell line stably expressing JellyOp and transiently transfecting it with the GloSensor cAMP reporter (Fig. 2a). Fibroblasts transfect less readily than HEK293 cells [21] (and note the difference in opsin expression between HEK293 (Fig. 3c) and rat1 fibroblasts (Fig. 4a)) and, as a result, the amount of GloSensor luminescence activity was low when compared with HEK293 cells. Nevertheless, JellyOp expressing cells responded to flashes of light with transient and repeatable increases in luminescence and to sustained illumination with a maintained response (Fig. 2a). As an additional confirmation of this effect we assayed cAMP directly using an ELISA. Two minutes of light exposure induced a 29-fold increase in intracellular cAMP concentration on average in rat1 cells expressing JellyOp but not in dark control cells (*P* < 0.05, 3.98 ± 3.39 μM to 116.58 ± 73.09 μM, JellyOp expressing cells, dark to post-light maximum, mean ± SEM, Student’s *t* test, n = 4).

To assess the impact of time-delimited activation of JellyOp on the clock, we tracked circadian rhythms in luminescence from the *per2::luc* reporter in rat1 fibroblasts expressing this photopigment. In accordance with the low dark activity of this pigment, the inclusion of JellyOp did not alter the period or amplitude of rhythms in luminescence (Fig. 2b). When JellyOp was activated by light exposure (5 s light pulses every 30 s over 4 h) large shifts in circadian phase could be observed in JellyOp expressing cells. Cells not expressing the opsin, by contrast, showed no response to light (Fig. 2b). The magnitude and sign of the shift in JellyOp expressing cells was strongly dependent upon the circadian phase at which it was applied (Fig. 2b). To describe this feature in more detail, we applied light pulses across the circadian cycle in order to describe a phase response curve (Fig. 2c; values in Additional file 1). This revealed strong phase advances between circadian time (CT) 0–4 (where by convention the peak of the *per2::luc* luminescence rhythm is designated CT12 [20, 22]), and phase delays between CT20 and CT24. In between these times, light had little impact on the clock.

### An arrestin-biased variant of JellyOp

The strong phase shifts induced by JellyOp activation confirm the ability of GPCR signalling to reset the circadian clock. Given the established phase resetting ability of drugs targeting cAMP, the most parsimonious explanation for those data is that they reflect the Gαs activating properties of JellyOp. However, they do not exclude a role for the additional arrestin-dependent signalling capacity of GPCRs. It is possible to substantially inhibit G-protein activation without abolishing arrestin binding by changing key residues near the conserved DRY motif (within the third transmembrane loop (TM3) and second intracellular loop (IC2)) of GPCRs, thus creating a functionally biased receptor [23–26]. We aimed to design a mutant of JellyOp that would still be capable of arrestin-dependent signalling but with reduced G-protein activation in order to dissect possible contributions of these pathways in resetting the circadian clock. In addition, we aimed to design a mutant with as few amino acid substitutions as possible to limit changes in structural stability. We therefore assessed the ability of a single point mutation F112A to inhibit JellyOp’s ability to increase cAMP in HEK293 cells (Additional file 2: Figure S1, Fig. 3a). We found that the light-induced increase in GloSensor luminescence was substantially reduced in cells expressing this mutant compared to the intact JellyOp. Luminescence values were much more similar to those measured from cells expressing a variant of JellyOp lacking a retinal binding lysine residue that is essential for opsin function [13] (K296A using bovine rhodopsin numbering; mean ± SEM max fold increase in RLU JellyOp 245.7 ± 20.3, JellyOp F112A 11.08 ± 2.84, JellyOp K296A 1.64 ± 0.25, n ≥ 3, one-way ANOVA with post-hoc Tukey’s test F112A vs. K296A *P* = 0.97; values in Additional file 1).

We first confirmed that the F112A mutation had genuinely reduced G-protein activation and not simply switched selectivity to another G-protein class. We assayed Gαq/11 activity using an aequorin luminescent reporter for intracellular calcium [27]. HEK293 cells expressing JellyOp F112A failed to produce a significant increase in the activity of this reporter (Fig. 3b; values in Additional file 1), indicating a lack of Gαq/11 activity. We also assessed Gαi/o signalling by including PTX in the GloSensor assay. Inhibition of Gαi/o with this toxin did not significantly enhance response magnitude to a flash of light (*P* = 0.12, peak response amplitude with PTX normalised to peak response amplitude without PTX: 1.12 ± 0.06, mean ± SEM, n = 5; values in Additional file 1) as would be expected if JellyOp F112A had measurable interaction with this G-protein class. Given the overall reduced G-protein activity of the mutant, we additionally confirmed that both JellyOp and JellyOp F112A are expressed well in these cells (Fig. 3c).

We then turned our attention to arrestin binding. We employed a split luciferase method to track JellyOp arrestin interactions. In brief, we tagged the C-terminus of an opsin with the C-terminal fragment of a click beetle luciferase coding sequence and appended the other portion of the luciferase to the N-terminus of either β-arrestin 1 or 2 (Fig. 3d) [28]. In this arrangement, binding of β-arrestin to an opsin is predicted to bring the two halves of the luciferase together, allowing them to form a functional catalytic unit. Thus, luminescence can provide a real-time read-out of opsin:β-arrestin interactions. We first checked whether melanopsin and rhodopsin, as positive controls, show measurable opsin:arrestin interactions with this method. Indeed, both melanopsin and rhodopsin showed increased interaction with β-arrestin 1 and 2 in the light (0.1 mW cm^–2^; Fig. 3e; n = 4; Student’s *t* test; values in Additional file 1). When applied to HEK293 cells with stable expression of either native JellyOp or F112A mutant, this split luciferase reporter revealed light-dependent binding of both β-arrestin 1 and 2 (Fig. 3f). To confirm that this was a specific event, we repeated the experiment without adding the chromophore and, as expected, the light-dependent arrestin interaction disappeared (Fig. 3f; values in Additional file 1). As differences in reporter activity in stable cell lines can reflect differences in opsin expression associated with integration site of the transgene, we next explored the ability of JellyOP and the F112A mutant to interact with β-arrestin 1 and 2 under transient transfection. Light-dependent interactions with both arrestins were apparent for both opsins and there was no suggestion of a difference in either response amplitude or kinetics between the pigments (Fig. 3g, h; n = 3; values in Additional file 1). The results confirmed that both JellyOp and JellyOp F112A interact with β-arrestin upon light stimulation.

### Arrestin-biased JellyOp F112A phase shifts the circadian clock

We next set out to determine whether the JellyOp F112A mutant could phase shift the fibroblast circadian clock. JellyOp F112A expressed well in rat1 *per2::luc* fibroblasts (Fig. 4a). We first confirmed that the F112A mutant had a similar impact on JellyOp F112A’s signalling in these cells as it had in HEK293 cells. We assessed light dependent Gαs activation in this cell line by measuring cAMP using an ELISA. No significant increase in intracellular cAMP concentration was seen following 2 min of light exposure in JellyOp F112A-expressing cells (4.36 ± 0.46 μM in the dark and 2.91 ± 1.01 μM following light, mean ± SEM, n = 3, *P* = 0.26, Student’s *t* test). We then confirmed that JellyOp F112A retained the light-dependent arrestin interaction. The poor transfection efficiency of rat1 cells was a practical barrier to employing the split luciferase approach in this cell line. Therefore, we instead turned to a proximity ligation assay, in which a polymerase reaction incorporates red fluorescent nucleotides when there is a close physical proximity between antibodies targeting JellyOp (a 1D4 epitope) and arrestin (both arrestin 1 and 2). Changes in the frequency of red pixels within the cell cytoplasm following 2 min of light exposure were normalised to changes in cells with no opsin. In three replicates, the increase in red pixels in cells expressing JellyOp compared to no opsin ranged between 3 and 34 pixels per cell and in JellyOp F112A-expressing cells between 12 to 58 pixels per cell. This confirmed a light-dependent increase in opsin:arrestin interactions in rat1 fibroblasts expressing JellyOp F112A.

Turning to the circadian oscillator, we found that light induced large shifts in rhythms of the *per2::luc* reporter in rat1 fibroblasts expressing JellyOp F112A (Fig. 4b, c). The magnitude of these shifts could be as large as those induced by the native pigment (the largest phase shift in hours, mean ± SEM, 6.96 ± 0.54 and 5.28 ± 1.40, for JellyOp and JellyOp F112A, respectively, unpaired *t* test *P* = 0.23; values in Additional file 1). However, the phase response curve for this mutant was advanced by approximately 5.6 h compared to that of JellyOp (x-intercept for linear regression = 5.57 vs. 11.34, for JellyOp F112A and JellyOp, respectively), with the result that JellyOp F112A activation induced large phase advances at times when JellyOp induced phase delays, and vice versa (Fig. 4c).

The difference in the light phase response curve between JellyOp- and JellyOp F112A-expressing cells is consistent with the view that the intracellular events linking receptor activation to the molecular clock are quite distinct in these two systems. The increase in cAMP induced by JellyOp is predicted to induce expression of *per2* and *cry2*, whose promoters contain cAMP response elements. This, in turn, is an accepted mechanism for shifting circadian phase. We therefore looked for light-dependent induction of clock gene expression in rat1 cells expressing JellyOp and JellyOp F112A using quantitative RT-PCR (Fig. 4d, e, values in Additional file 1). We found a significant increase in expression of *cry2* in JellyOp-expressing cells at 30 and 120 min, and *per2* at 120 min after a light pulse (one-sample *t* test, *P* < 0.05, Fig. 4d). By contrast, there was no significant change in expression of either of these clock genes in JellyOp F112A cells (Fig. 4e).

## Discussion

We show here that both native JellyOp and a structural variant (JellyOp F112A), with impaired G-protein signalling but retaining light-dependent arrestin binding, can phase shift the circadian clock. The conclusion that they do so by separable mechanisms is supported not only by our characterisation of their relative signalling capabilities, but also by differences in the circadian phase at which they have their biggest impact and by our demonstration that, while JellyOp induces expression of the clock genes *per2* and *cry2* (an established mechanism of shifting the clock), JellyOp F112A does not.

Our characterisation of JellyOp F112A indicates that the replacement of phenylalanine with a non-polar single amino acid at this site is sufficient to abolish G-protein signalling, without interfering with light activation or subsequent β-arrestin binding in this opsin. Sequence alignments predict that the F112 residue will lie around the transition from TM3 and the second intracellular loop (Additional file 2: Figure S1). Our data are thus consistent with the view that this domain plays a key role in G-protein interaction [29]. The appearance of phenylalanine in this position is highly conserved across GPCRs, and its replacement by alanine has previously been shown to inhibit G-protein interaction [23, 24].

JellyOp and JellyOp F112A represent attractive tools for optogenetic control over Gαs and β-arrestin signalling. JellyOp is the only opsin known to be naturally Gαs coupled; however, optogenetic control over this signalling pathway has also been attained using an ‘Opto-β2AR’ chimera between rod opsin and the β2-adrenergic receptor [17, 18, 30]. Here, we extend our previous side-by-side comparison of these two approaches [13] and confirm that JellyOp has several advantages. In the dark, JellyOp expressing cells do not show elevated levels of cAMP compared to untransfected controls, whereas cells expressing Opto-hβ_2_AR appear to have significant constitutive activity. The subsequent magnitude of the light response is significantly larger for JellyOp and can be achieved using less powerful light. In addition, we find that the Opto-hβ_2_AR has measurable Gαi activity making it less selective for Gαs than JellyOp. An equivalent comparison between JellyOp F112A and a recently published arrestin-biased version of the Opto-hβ_2_AR carrying mutations at three residues on the intracellular surface [30] is currently lacking. However, Peterson et al. [24] found that, while arrestin bias could be achieved in the dopamine D2 receptor by replacing either the single residue at the equivalent site to JellyOp F112 plus one other, or the three TYY residues applied to the Opto-hβ_2_AR, the latter led to protein instability and less robust separation of G-protein and β-arrestin interactions.

The split luciferase reporter for β-arrestin interactions affords a new approach to interrogating opsin:β-arrestin interactions that is uniquely suitable for photopigments. This assay lacks the spatial and temporal resolution of FRET-based reporters, but has the advantage of reporting activity more quantitatively. Firstly, a fluorescent protein pair, CFP and YFP, is used for FRET experiments to detect the dynamic changes of GPCR activity [31]. However, this becomes a problem when assessing opsin activity, as the blue light used to excite CFP should also activate and bleach the opsins, complicating the accurate quantification of their activity [32]. In contrast, bioluminescence-based reporters, including the split luciferase probe, do not require any excitation light and emit many fewer photons than fluorescent reporters do. Therefore, the assays with bioluminescence probes should perturb the activity of opsins during the measurement much less, providing more accurate results. Secondly, this assay provides a high signal-to-noise ratio due to almost negligible background luminescence, which is also advantageous for quantitative detection [33]. Finally, considering that the bioluminescence response of the split luciferase probe occurs on the order of 1 min [28], the probe can have enough temporal resolution to monitor the dynamic patterns of opsin-β-arrestin interaction, which holds on the order of 10 min [34]. Therefore, the split luciferase reporter is a suitable tool to quantitatively detect the dynamics of protein interactions in living cells without interfering with the photoreactive proteins.

While the importance of G-protein coupled receptors in entraining circadian clocks is well established, to our knowledge, this study is the first to implicate β-arrestin signalling in this process. Activation of the β-arrestin-biased opioid receptor CXCR7 has been shown to enhance the amplitude of circadian rhythms in adrenal glucocorticoid production in mice, but not via effects on the clock mechanism itself [35]. Application of a mutated version of JellyOp lacking arrestin binding is a conceptually attractive approach to confirming the β-arrestin link to the circadian clock. In practice, however, it would be hard to interpret the outcome of such an experiment as any interruption to β-arrestin binding would also interfere with termination of G-protein signaling, which could itself alter the clock response. Nevertheless, several aspects of our data provide confidence that JellyOp and JellyOp F112A interact with the clock via fundamentally different mechanisms. Thus, phase shifts produced by these two pigments occur at different circadian phases, and have differing effects on clock gene expression. This indicates that the phase resetting ability of JellyOp F112A does not reflect residual G-protein signalling activity, leaving its demonstrated arrestin-interaction activity as the most parsimonious origin of the phase shift.

Future work will be required to establish the signalling steps linking JellyOp F112A to the circadian machinery. β-arrestin activation has been linked to influential signalling cascades via interactions with MAP kinases, PI3K/AKt and RhoA [8, 9]. Several of these processes have been linked to the molecular clock (MAPK directly [36] or via mTOR [37–39], AKT [39] and GSK3 [40–43]). One important aspect of our data is that JellyOp F112A produced very high amplitude shifts without detectable changes in *per2* or *cry2* expression. This implies that it interacts with the clock machinery via a post-transcriptional mechanism. The translation, cellular localisation and longevity of clock proteins are all under dynamic control and provide opportunities to influence the period or phase of the oscillator [44, 45].

The existence of separable G-protein-dependent and -independent pathways linking GPCRs to the circadian machinery allows additional flexibility for the circadian system. The degree to which GPCRs activate G-protein and/or arrestin pathways can differ between receptors for the same ligand. It also can vary for the same receptor depending upon the ligand bound and the rhodopsin kinase it recruits upon activation [9, 10]. In this way, having separable G-protein and arrestin pathways provides a mechanism for the same signalling molecule to activate different pathways, and for different molecules to activate different pathways via the same receptor. In the context of the multi-oscillator circadian system, these effects could provide a mechanism for the different tissue oscillators to respond appropriately to environmental signals. Under stable entrainment, the phase of the molecular clockwork differs between tissues and this effect can be exacerbated by timed feeding [46–48] or scheduled exercise [49, 50]. If, as this implies, flexibility in the relative phasing of tissue clocks helps organisms tailor physiological rhythms to environmental conditions, then having separable G-protein and arrestin signalling routes could allow cells to adopt quite different phases to the same signalling molecule depending upon their tissue location and/or physiological state. Another possibility is that the dual signalling function of GPCRs allows additional scope for the central clock to influence the response of peripheral clocks to environmental signals. Thus, some of the kinase cascades targeted by β-arrestin are also thought to provide a route for nutrient levels to entrain the clock (e.g. TOR/AKT nutrient sensing [51], glucose GSK3 [52]). Activation of an arrestin-biased GPCR could thus either augment or inhibit the phase shifting effect of a nutrient signal.

## Conclusions

We show here that both native JellyOp and JellyOp F112A support light-dependent shifts in the fibroblast circadian clock, but do so at different circadian phases and via different molecular mechanisms. These experiments thus reveal separable G-protein and arrestin pathways through which GPCRs can entrain circadian clocks. The pair of optogenetic tools developed herein are suitable for manipulating Gαs- and β-arrestin-biased signalling in live cells.

## Methods

### Cell culture

HEK293 (ATCC) and rat fibroblast cells (kindly donated by Dr Qing-Jun Meng, University of Manchester) were propagated at 37 °C in Dulbecco’s modified Eagle’s medium, 4.5 g L^–1^ D-glucose, sodium pyruvate and L-glutamine (Sigma) with 10% foetal bovine serum (FBS; Sigma) and penicillin/streptomycin in a 5% CO_2_ atmosphere. Rat1 fibroblast cells were additionally maintained with 100 μg mL^–1^ hygromycin (Invivogen).

### Construction of expression vectors

A mammalian expression plasmid pcDNA5 GloSensor 22 F containing the open reading frames of a GloSensor cAMP reporter (Promega Corp) was used to report cAMP levels in HEK293 cells, as described elsewhere [13, 27]. The plasmid pGloSensor 20 F (Promega Corp) was used to report cAMP levels in rat1 fibroblasts. The plasmid pcDNA5 mtAeq was used to express a mitochondrially targeted aequorin protein in order to report intracellular Ca^2+^ changes in HEK293 cells as a function of luminescence [26]. Mammalian expression plasmids containing the open reading frames of the Opto-hβ_2_AR Rh1B2AR 1-t, bovine rhodopsin and JellyOp were used as previously described (pcDNA3 Rh1B2AR 1-t and pcDNA3 JellyOp [13] and pcDNA3 Rh1 [27]). The open reading frame of JellyOp (Genbank AB435549) was also excised from a cloning vector puc57 JellyOp [13] and ligated into the mammalian expression vector pIRES-AcGFP. Site directed mutagenesis of pcDNA3 JellyOp and pIRES-JellyOp-AcGFP was carried out with primers designed to alter nucleotide triplets equating to amino acid site 112 (JellyOp residue numbering) from TTC to GCC and also amino acid site 275 (this is equivalent to site 296 in bovine rhodopsin; Additional file 2: Figure S1). Mutagenesis was carried out using a Quikchange Lightening site-directed mutagenesis kit (Agilent Technologies, USA) according to the manufacturer’s instructions. All plasmids were sequenced by the University of Manchester DNA sequencing facility and verified prior to use.

For split luciferase experiments the following plasmids were used. The open reading frames of the wild type and the mutant JellyOp were inserted into the mammalian expression vector pcDNA4_ELucC394-542 to form pc4_JellyOp-ElucC as described in a previous report [53]. Mammalian expression plasmids containing the open reading frames of the β-arrestin and the N-terminal fragment of the luciferase (Eluc1-415) were used as previously described (pc3.1_ELucN-ARRB2) [53]. The plasmid sequences of JellyOp-ElucC were analyzed and verified by the Eurofins Sequencing Service.

### Luminescent second messenger assays in HEK293 cells

Cells were transiently transfected with reporters and opsins prior to assays using lipofectamine 2000 (Thermo Fisher Scientific) for 4–6 h and incubated overnight with 10 μM 9-*cis* retinal (Sigma-Aldrich). Gαs activity was assessed in cells by measuring their luminescent output with a Fluostar Optima plate reader (BMG Labtech, Germany) as described in detail elsewhere [27]. The following morning, cells were incubated for 2 h at room temperature with an additional 2 mM final concentration of beetle luciferin potassium salt (Promega), reconstituted in 10 mM HEPES buffer in L-15 media with 1% FBS. Cell plates were then placed into a plate reader with a photomultiplier tube and luminescence recorded every 30s. All recordings were performed at 21–25 °C. Cells were exposed to either a camera flash bulb (The Jessop Group Ltd., UK; calculated to deliver at least 3000 Wcm^–2^ with a duration of less than 2 ms following measurement with a photodiode and calibrated against the voltage produced by known light power outputs), a xenon-ARC lamp for 2 s (broad spectrum with no filters; typical irradiance 2 mW cm^–2^ at the level of the cells) or a blue LED for 5 s (λ_max_ = 470 nm). Any light-dependent increase in cAMP reporter activity was assumed to be a result of Gαs activity as JellyOp is known to activate Gαs [13, 14]. For probing the Gαi activity in predominately Gαs coupled receptors such as JellyOp, cells were incubated with 10 μM pertussis toxin overnight (PTX, Sigma), known to specifically block Gαi activity. Experiments were carried out as described above, with any resulting difference in luminescence between PTX- and non-PTX-treated wells interpreted as being a result of blocked Gαi activity.

Cells transfected with pcDNA5/FRT/TO mtAeq and opsins were used to measure calcium-dependent changes in luminescence, which would be expected from a Gαq-coupled opsin, as described previously [27]. Following transfection, cells were incubated with 10 μM Coelenterazine *h* (Biotium) for 2 h in the dark before recording luminescence on a plate reader. Sample cells without opsins were treated with 100 μM carbachol (Sigma) as a positive control for increases in intracellular calcium.

### Irradiance response curves

Cells expressing either Opto-hβ_2_AR or JellyOp were prepared as described above for luminescent second messenger assays. For irradiance response curves, cells transiently expressing Opto-hβ_2_AR or JellyOp and GloSensor 22 F were stimulated simultaneously using a custom built 32 LED array (λ_max_ = 470 nm) that was attached on top of the experimental plate once removed from the plate reader. Each of eight columns of four wells was randomly assigned one of eight intensities of light spanning three orders of magnitude. Response per well was determined as the max RLU value following 2 s light stimulation, normalised to the mean baseline RLU of 10 cycles before light stimulation. Data was fitted with sigmoidal dose-response curves (variable slope, not restrained) using GraphPad Prism, from which EP_50_ values were obtained.

### Rat1 cell line construction

*Per2::luc* rat1 fibroblasts, which express a minimal promoter of the mouse *per2* gene fused to a luciferase gene (kindly donated by Qing-Jun Meng, University of Manchester), were transfected with ApaLI-linearised pIRES-JellyOp-AcGFP or pIRES-JellyOpF112A-AcGFP using Lipofectamine 2000 (Life Technologies). Polyclonal colonies or isogenic colonies isolated with cloning rings (Sigma-Aldrich) were then maintained with 200 μg mL^–1^ neomycin (InvivoGen) and passaged in dim red light.

Stable *per2::luc* fibroblasts expressing JellyOp or JellyOp F112A were seeded in 96-well solid white plates as for HEK293 luminescent second messenger assays in triplicate. Cells were transiently transfected with pGloSensor 20 F (Promega) with Lipofectamine 2000 as per the manufacturer’s instructions. Media was replaced after 6 h and cells were incubated overnight in L-15 (Life Technologies) with 10% foetal calf serum and 10 μM 9-*cis* retinal at 37 °C before transferring the 96-well plate to the Fluostar luminescent reader for cAMP reporter measurements as previously described. A camera flash bulb (The Jessop Group Ltd.) was used as the light source for stimulating these cells, before placing the plate back into the reader for further luminescent readings.

### Immunocytochemical labelling of cells

All opsin plasmids manufactured for this work incorporated a 1D4 epitope fused to the C-terminal (TESTQVAPA). A total of 1 × 10^5^ *per2::luc* JellyOp and JellyOp F112A rat1 fibroblasts or a HEK293 cell line (FLP-IN-293 GloSensor 20 F) previously constructed in our lab [13] were seeded (in dim red light) onto glass coverslips in a 24-well plate (Corning, USA). HEK293 cells were transiently transfected with pIRES JellyOp-AcGFP or pcDNA3 JellyOpF112A-AcGFP with Lipofectamine 2000, as described above. All cells were then incubated with DMEM (without phenol red)/FBS with 10 μM 9-*cis* retinal (Sigma-Aldrich) overnight. Cells were fixed with 4% paraformaldehyde (Sigma-Aldrich) in 1× phosphate buffer and blocked. Cells were incubated with 1:500 1D4 antibody (Abcam, cat#Ab5417, lot: GR272982-11, RRID: AB_304874), then with 1:1000 Alexa Fluor 594 (conjugated anti-mouse secondary antibody (Invitrogen cat#A-21203, lot:1722995, RRID: AB_141633). Cells were mounted with Vectashield with DAPI (Vectalabs). Rat1 fibroblasts were visualised on a Delta Vision deconvolution microscope (Applied Precision, USA) using a 60× Plan Apo objective magnification. The images were collected using a Coolsnap HQ (Photometrics, USA) camera with a Z optical spacing of 0.35 μm and deconvolved using the Softworx software (Applied Systems). HEK293 cells were examined on an Olympus BX51 upright microscope using a 20× Plan Fln objective and captured using a Coolsnap ES camera (Photometrics, Tucson, AZ) through MetaVue Software (Molecular Devices Ltd. Wokingham, UK). Images were taken under specific band pass filter sets and colour-combined images were used.

### Split luciferase complementation assays for binding of opsin to β-arrestin in HEK293 cells

Luminescence measurements on 96-well plates were performed according to previous reports [28]. In case of transient expression, HEK293 cells stably expressing ElucN-arrestin were seeded on 96-well plates (2.0 × 10^5^ cells/mL, 100 μL/well) 24 h before the transfection of pcDNA4 opsin-ElucC. In case of the light-dose analysis, HEK293 cells stably expressing opsin-ElucC and ElucN-arrestin were seeded with a cell density of 2.0 × 10^5^ cells/mL. The medium was replaced to phenol red-free medium supplemented with 1% FBS and 10 μM 9-*cis* retinal 48 h after the seeding. The cells were incubated for 1 h at 37 °C in 5% CO_2_ and stimulated under white room light (0.1 mW cm^–2^ at the level of plates). The medium was removed and 100 μL of a reagent (Emerald Luc Luciferase Assay Reagent, Toyobo, Japan) was added to each well. Luminescence from the wells was obtained using a luminescence microtiter plate reader (TriStar LB941, Berthold). In the apparatus, the plates were first gently shaken for 5 min, and then luminescence intensities were measured.

### cAMP ELISA

Stable cells from the rat1 fibroblast lines *per2::luc*, *per2::luc* JellyOp and *per2::luc* JellyOp F112A were plated at 4 × 10^4^ cells mL^–1^ in 12-well plates and left overnight at 37 °C with 5% CO_2_ in DMEM lacking phenol red and supplemented with 10% foetal calf serum and 10 M 9-*cis* retinal. Cells were either left in the dark (control) for 2 min or exposed to a flash from the camera flash bulb, as described above. All cells were then lysed with 0.1 μM HCl in the dark for 10 min. Cell lysates were centrifuged and the supernatant was used in a direct cAMP enzyme-linked immunoassay kit (Sigma Aldrich) according to manufacturer instructions. The protein content was determined with a Fluka protein quantification kit (Sigma-Aldrich) and samples were diluted as necessary to ensure equal protein content before being used in ELISA. Optical measurements were taken on a Fluostar Optima plate reader (BMG Labtech).

### Proximity ligation assay of opsin and arrestin in rat1 fibroblasts

Rat1 fibroblasts of lines *per2::luc*, *per2::luc* JellyOp and *per2::luc* JellyOp F112A were plated and the media replaced as for immunocytochemistry, as described above. Allocated light-exposed cells were removed from the incubator and placed on top of a litebook Elite white light source for 2 min (The Litebook Company Ltd., Canada; 2.6 mW cm^–2^ at the level of the cells). Cells were washed with PBS and fixed in 4% paraformaldehyde for 30 min. A Duolink protocol was then followed using a Duolink anti-mouse MINUS/anti-rabbit PLUS red kit (Olink Bioscience) with antibodies for pan-arrestin (ab2914; Abcam PLC) and anti-rhodopsin (1D4; Pierce/Thermo Scientific Fisher Inc.). Labelled cells were mounted in Prolong Gold with DAPI (Life Technologies). For each treatment and sample a minimum of 10 photomicrographs were taken at random intervals with an Olympus BX51 microscope with band pass filter sets specific for Texas Red and DAPI and a coolsnap ES camera (Photometrics) with Metavue software (Molecular Devices; Bioimaging Facility, The University of Manchester). Photomicrographs of the red channel only were converted top black and white and the brightness threshold set to 170. Fluorescent signal should only be present when the two probes are in close proximity, indicating functional activity. The number of pixels above the threshold was divided by the number of DAPI-positive nuclei in blue-channel photomicrographs.

### Bioluminescence recording of *per2::luc* fibroblasts

The recording medium for bioluminescence recordings of fibroblast cultures consisted of high-glucose, high-glutamine, no phenol red 10 g L^–1^ DMEM supplemented with 3.5 g L^–1^ D- glucose (Sigma-Aldrich), 350 mg mL^–1^ sodium bicarbonate (Sigma-Aldrich), 10 mM Hepes buffer, 2% B27 supplement (Life Technologies), 20 U mL^–1^ penicillin and 25 μg mL^–1^ streptomycin (Sigma-Aldrich), 100 μM beetle luciferin (Promega) and 10 μM 9-cis retinal. Prior to recording, cells were synchronised with 200 nM dexamethasone (Sigma-Aldrich) for 1 h, after which the medium was replaced with the recording medium. Cell dishes were then sealed with glass coverslips using high vacuum grease (Dow Corning) and bioluminescence recordings were conducted within a Lumicycle machine (Actimetrics) housed in a 37 °C incubator. The photon count was sampled from each well with 75 s resolution at 10-min intervals. Data for approximately the first 24 h in culture were not used for further analysis in order to allow the cultures to settle, light pulses (4 h of 5 s light steps every 30 s using white light from a Fiber-Lite® DC950 Illuminator (Dolan-Jenner Industries) that was fed into the incubator through a liquid light guide (Knight photonics) with a UV and infrared cut-off and controlled via a programmable shutter (Cairn)) were applied after at least two subsequent peaks of *per2::luc* luminescence. Irradiance at the level of the cells was 28.40 mW cm^–2^. Circadian time of pulses was calculated using a Lumicycle inbuilt analysis tool. Phase shifts were estimated from raw data exported to Microsoft Excel (Microsoft). Traces were detrended by subtracting the 24-h running average to filter out baseline drift in the *per2::luc* rhythm, and smoothed to minimise the impact of high frequency fluctuations using a 2-h running average. The troughs and peaks of the fibroblast rhythm were assigned as CT0 and CT12, respectively. To measure light-induced phase responses in the *per2::luc* rhythm, the unperturbed rhythm prior to treatment was fit with a continuous sine wave using Clockwise curve fitting software [54, 55]. This sine wave was extrapolated to the days after the pulse as an indication of the projected phase of an unperturbed rhythm. Assigning the peaks as reference markers, the phase shift was scored as the difference in the time of the observed peak of luminescence and that predicted by the projected sine wave on day 2 after the light pulse. The phase shifts were plotted as a function of the circadian time at which the light pulse was delivered. Phase shifting experiments for control *per2::luc*, JellyOP and JellyOP F112A fibroblast cultures were performed in parallel using single starting batches of stably transfected lines. GraphPad was used for linear regression in phase response profiles for JellyOp and JellyOp F112A.

### qPCR

Rat1 fibroblasts were exposed to 30 min or 2 h intermittent (5 s pulses every 30s), infra-red and ultraviolet filtered bright white light (28.40 mW cm^–2^) at different circadian times, namely at CT2 and CT20 for JellyOp and JellyOp F112A, respectively. Total RNA was extracted from all samples, including dark controls, using NucleoSpin RNA (Macherey Nagel). Following DNase treatment with RQ1 RNase-free DNase (Promega), RT-PCR was performed using the High Capacity RNA-to-cDNA kit (Life Technologies). qPCR was performed with SYBER GREEN master mix with a Light Cycler® 480 (Roche). All qPCR results were normalised to actin level and to additional standards. Each sample was run in triplicate and the data was analysed using the 2^−ΔΔCT^ method [56].

Primers that were used were previously published: per2 and rev-erba [57] and cry2 [58].

## Additional files

Additional file 1: Supporting data file. (XLSX 2222 kb)

Additional file 2: Figure S1. An amino acid sequence alignment of JellyOp WT (Genbank AB435549) from squid rhodopsin (X70498.1) and bovine rhodopsin (NM_000024). Boundaries of the transmembrane regions are highlighted in grey. The lysine residue in TM7, which forms a Schiff-base linkage with the retinaldehyde chromophore, is boxed. The JellyOp residue F112, which is substituted for alanine in the JellyOp F112A mutant, is highlighted in green [59, 60]. (TIF 49066 kb)
