# Supplementary figures and images for: Optogenetic interrogation reveals separable G-protein-dependent and -independent signalling linking G-protein-coupled receptors to the circadian oscillator
Source: BMC Biol. 2017 May 15;15:40. doi: 10.1186/s12915-017-0380-8 (PMC5430609; doi:10.1186/s12915-017-0380-8)

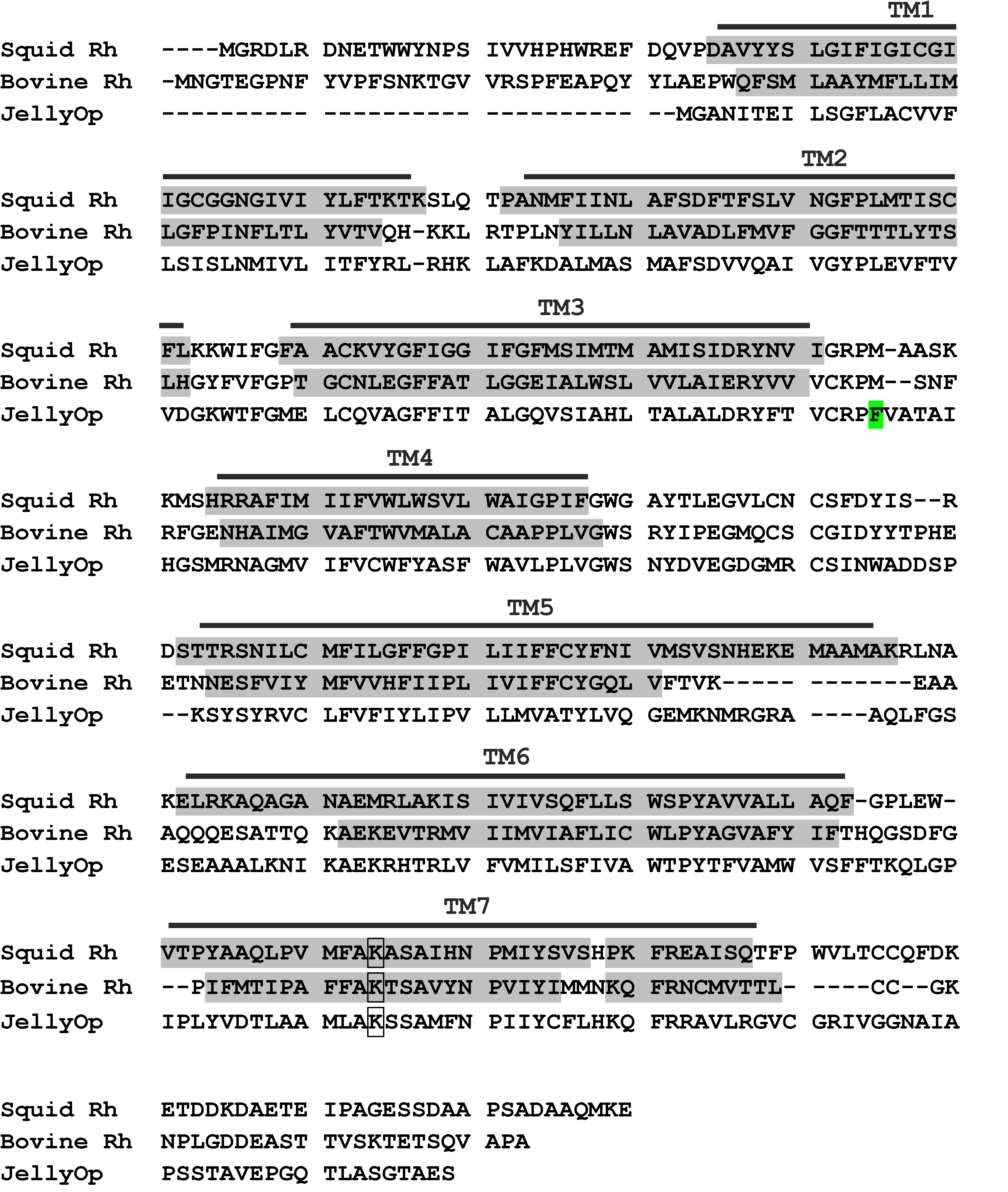

Supplement: Supplementary file 2 — An amino acid sequence alignment of JellyOp WT (Genbank AB435549) from squid rhodopsin (X70498.1) and bovine rhodopsin (NM_000024). Boundaries of the transmembrane regions are highlighted in grey. The lysine residue in TM7, which forms a Schiff-base linkage with the retinaldehyde chromophore, is boxed. The JellyOp residue F112, which is substituted for alanine in the JellyOp F112A mutant, is highlighted in green [59, 60]. (TIF 49066 kb) [file 12915_2017_380_MOESM2_ESM.tif]
